# Reliability constrained dynamic generation expansion planning using honey badger algorithm

**DOI:** 10.1038/s41598-023-43622-9

**Published:** 2023-10-05

**Authors:** Adel A. Abou El Ela, Ragab A. El-Sehiemy, Abdullah M. Shaheen, Ayman S. Shalaby, Mohamed T. Mouafi

**Affiliations:** 1 Electrical Engineering Department, Faculty of Engineering, Menoufiya University, Shibîn el Kôm, Egypt; 2 Electrical Engineering Department, Faculty of Engineering, Kafrelsheikh University, Kafr El Sheikh, Egypt; 3 Electrical Engineering Department, Faculty of Engineering, Suez University, El Suweis, Egypt; 4 Middle Delta Electricity Production Company (MDEPCo), Talkha, Egypt

**Keywords:** Energy science and technology, Engineering, Electrical and electronic engineering

## Abstract

Generation expansion planning (GEP) is a complex, highly constrained, non-linear, discrete and dynamic optimization task aimed at determining the optimum generation technology mix of the best expansion alternative for long-term planning horizon. This paper presents a new framework to study the GEP in a multi-stage horizon with reliability constrained. GEP problem is presented to minimize the capital investment costs, salvage value cost, operation and maintenance, and outage cost under several constraints over planning horizon. Added to that, the spinning reserve, fuel mix ratio and reliability in terms of Loss of Load Probability are maintained. Moreover, to decrease the GEP problem search space and reduce the computational time, some modifications are proposed such as the Virtual mapping procedure, penalty factor approach, and the modified of intelligent initial population generation. For solving the proposed reliability constrained GEP problem, a novel honey badger algorithm (HBA) is developed. It is a meta-heuristic search algorithm inspired from the intelligent foraging behavior of honey badger to reach its prey. In HBA, the dynamic search behavior of honey badger with digging and honey finding approaches is formulated into exploration and exploitation phases. Added to that, several modern meta-heuristic optimization algorithms are employed which are crow search algorithm, aquila optimizer, bald eagle search and particle swarm optimization. These algorithms are applied, in a comparative manner, for three test case studies for 6-year, 12-year, and 24-year of short- and long-term planning horizon having five types of candidate units. The obtained results by all these proposed algorithms are compared and validated the effectiveness and superiority of the HBA over the other applied algorithms.

## Introduction

The power system is composed of three main sectors which are generation sector, transmission sector, and distribution sector. The planning issue of each element is a process that the investment decisions are made based on different aims to meet the forecast demand^1^. Generation expansion planning (GEP) is an important decision-making activity in power systems which aims at deciding on new as well as upgrading existing generation units with different types. The decision should determine the features of where to allocate, when to install the new generation units, and what to select the new generation technology over a long-range planning horizon. The main objective function of GEP is to minimize the total costs which include the investment, operation and maintenance costs, and outage costs. At the same time, the upper construction limit, reliability, fuel mix, reserve margin constraints must be satisfied. GEP is a highly complex optimization problem due to including different nonlinear constraints. Therefore, the solution for this problem should be executed by more efficient methods to reach optimum solution^2,3^. GEP models are divided into two classes of static and dynamic. The static GEP model is a single period GEP problem which introduces less computational complexities of the problem, whereas dynamic GEP is a multi-period planning problem with higher computational complexity. Thus, dynamic GEP is more complex than static model.

### Literature review

A few contemporary review publications are available that categorize the GEP models^4–6^. The GEP optimization frameworks with incorporation of renewable energy divides the various approaches into four categories: (a) conventional approaches that incorporate environmental limitations throughout the GEP; (b) formulating the GEP as a multiple-objective optimization problem; (c) strategies for addressing variable RES-related uncertainties in the GEP process; and (d) different dynamics and issues brought about in the power networks due to the growing incorporation of intermittent RES. It contributes to an improved comprehension of the anticipated results of each technique by offering insights into the traits, benefits, and drawbacks of the theoretical methods used as well as their applicability for various parts of the problem. Because transmission network operators as well as other managers believe that such models produce reliable results, the list of national/regional optimization models is lengthy.

In generating system analysis, the ability to meet the demand requirements is measured by the reliability criterion of the system which depends on the availability of generation units. The reliability of a system can be calculated by different indices such as loss of load probability (LOLP), loss of load expectation (LOLE), and expected energy not served (EENS). LOLE index is widely used, because of its simplicity. EENS reflects the true risk than LOLE so that it is an appealing index than LOLE. Also, the EENS index can be used to calculate the expected energy produced by each generation unit for each stage of the planning horizon^3,7,8^. There are several tools to calculate the expected energy produced based on EENS. Therefore, the proper valuation of EENS is required to provide accurate estimates of the variable costs. Power system probabilistic production simulation (PPS) provides an accurate evaluation framework for EENS, and LOLP^3,9^. Also, several methods have been handled to solve such complicated optimization problem. Some of the classic optimization methods, that have applied to solve the GEP problem, include Analytic Hierarchy Process^10^, dynamic and quadratic programming^11,12^ and mixed integer nonlinear programming (MINLP) model^13,14^. In^14^, the option of Demand-Side Management (DSM) has been modeled as an equivalent generating unit which used only at peak load to maintain system reliability. In^15^, the GEP problem incorporating renewable energy has been presented where an hourly unit commitment problem has been used for supporting the selection of fuel mixes of power plants. In^16,17^, the least cost GEP model considering emission reduction has been presented that the penalty cost of emission was added to the objective function but the reliability constraint has not taken in account. Also, the GEP problem with the integration of renewable energy technologies and expected impacts in term of emission reduction has been presented in^18^. Despite the simple and easy implantation of MILP in solving the GEP problem, it has been utilized with low control variables. In^19^, MILP with fuzzy objectives has been applied to create the expansion model of the primary heat source in the district heating systems, combined heat and power units. In^20^, the GEP problem was handled by MILP considering the impact of unit commitment and renewable sources but the forced outage rate of generation units didn’t taken into account at expected energy produced calculation. However, because of GEP is a complex problem with nonlinear constraints, the convergence of those classical methods to the optimal decision makes difficult to solve. Also, there are commercial packages like Wien Automatic System Planning (WASP) Package has been used for solving GEP problem^21,22^. On the other side, the unavailability of generation units has not considered at variable costs calculations in^21^.

In addition to that, a mixed integer linear programming model for the optimal GEP problem was introduced in Ref.^23^. This model was designed as a two-step process, with the first phase including dynamic programming to determine the best connection lines for electricity and the subsequent phase involving optimal transmission and GEP. A dynamic, integrated planning model for GEP involving transmission expansions was additionally created in^24^ using the linearized form of the AC power flow model as the basis for the generalized bender's decomposition approach. With the goals of minimizing risks at each level of expected return and maximizing anticipated return for any particular level of risk, Ref.^25^ adapted portfolio theory to the optimum GEP issue of Iran including financial risk management. Furthermore, with the aim of minimizing the projected cost and the conditional value-at-risk, Jin et al. presented a two-phase stochastic mixed-integer programming to handle the GEP issue under unpredictable growth in demand and fuel prices development^26^. Ref.^27^ explores the complementing possibilities of demand-side response by looking at researches on the German subsequent energy system that would exclusively use RES. In the example of the Chilean electrical grid, Ref.^28^ handles the GEP concerning substantial RES penetration by using a column generation technique and a unique Dantzig-Wolfe decomposition. The study shows that the suggested method beats commercial solver software because it surpasses intractability and drastically lowers computing load.

Over the last decades, there has been a growing concern in modern nature-inspired algorithms which are classified as meta-heuristic algorithms such as genetic algorithm (GA)^29–31^, gravitational search algorithm (GSA)^32^, shuffled frog leaping algorithm (SFLA)^33^, and modified shuffled frog leaping algorithm (MSFLA)^9^. The performance of GA was improved by application of Virtual mapping procedure, and controlled elitism (NSGA-II) which has been used for solving the GEP problem with and without network constraints considered as in^29,31^, respectively and compared with differential evaluation as in^34^. Also, investigation the impact of RES penetration on environmental aspect has been conducted over the GEP model using GA^32^. The implantation of capacity expansion planning based on electricity market was developed in^30^ to help the generation companies to take their decision whether to invest on new assets. In^9^, the comparison between GA, SFLA, and MSFLA has been presented to solve GEP problem with reliability constrained. Also, particle swarm optimization (PSO), differential evolution (DE), evolutionary programming (EP), ant colony optimization (ACO), tabu search (TS), simulated annealing (SA), and hybrid approach have been applied in^35^. In this study, the penalty factor of reliability criteria, which were used, was very small compared to the objective function which doesn’t guarantee the feasibility of the achieved solutions. So that, the output results haven’t been achieved the reliability constraint. Thus, the value of the penalty factor must be equal to several times of the objective function. PSO algorithm has been used in^36^ to solve the GEP problem in the deregulated electricity market. Also, a centralized GEP problem has been addressed by a distributional robust chance-constrained as in^37^.

The GEP dimensionality is large which the length of a string is equal to the product of the number of planning stages and the number of candidate unit’s types. So that the Virtual mapping procedure (VMP), penalty factor approach (PFA), and the modified of intelligent initial population generation (MIIPG) have been accomplished to decrease the GEP problem search space and reduce the computational time^2,33^.

### Paper contributions

The contributions of this paper with respect to the previous research in the area can be summarized as follows:

- A multi-stage GEP with reliability constrained is presented to minimize the capital investment costs, salvage value cost, operation and maintenance, and outage cost under several constraints.
- A comparative assessment for solving the proposed reliability constrained GEP problem is performed of different modern algorithms of PSO, CSA, AO, BES, and HBA.
- Several modifications are proposed such as VMP, PFA, and MIIPG to decrease the GEP problem search space and reduce the computational time.
- A high effectiveness and superiority of the proposed HBA over the other applied algorithms for solving the proposed reliability constrained GEP problem for three test case studies for 3-year, 12-year, and 24-year of short- and long-term planning horizon.

### Paper organization

The rest of this paper is organized as follows: in the next section, the GEP problem formulation is presented. In “Honey badger algorithm for reliability constrained dynamic GEP” section, the GEP problem modifications are described, the proposed HBA algorithm for solving the reliability GEP problem is described in “Test system description and results discussion” section. The test system and simulation results for the reliability GEP problem are presented in “Conclusions” section. Finally, the paper is concluded in section VI.

## Formulation of dynamic generation expansion planning

### Objective function

Solving a reliability constrained GEP problem with minimum total costs aims at determining the optimum expansion plan over planning period that achieves minimum total cost under constraints satisfaction. The GEP total costs can be divided into two main parts which are related to new installed and already existed candidate units. In this context, the investment and salvage costs belong to the first part while operation and maintenance and outage costs are related to the candidate and existed units all together as following^9,33^:

#### Capital investment cost

This term represents the investment cost of new candidate units $I\left( {u_{t} } \right)$ which can be given by:

1 $$ I\left( {u_{t} } \right) = \left( {1 + i} \right)^{ - tc} \times \mathop \sum \limits_{k = 1}^{N} \left( {CI_{k} \times u_{t,k} } \right) $$

2 $$ tc = t_{o} + s \times \left( {t - 1} \right) $$

where, $i$ is the discount rate;$CI_{k}$ is the investment cost of each new candidate unit *k* added in stage *t* which are selected of different technologies; $N$ is the number of selected candidate units of technology k;$u_{t}$ is the capacity vector of alcandidate unit types in the stage;$t_{o}$ is the number of years between the reference date for discounting and the 1st year of study; $s$ is the number of years in each stage*t*.

#### Salvage value cost

Salvation value $SV\left( {u_{t} } \right)$ is the real value of generating unit at a specific time and after considering the depreciation rate which is calculated as following:

3 $$ SV\left( {u_{t} } \right) = \left( {1 + i} \right)^{ - Ts} \times \mathop \sum \limits_{k = 1}^{N} \left( {\delta_{k,t} \times CI_{k} \times u_{t,k} } \right) $$

4 $$ Ts = t_{o} + s \times T $$

where, $\delta_{k,t}$ is the salvage factor of unit k added in stage t;$T$ is the number of stages in the planning horizon. It is assumed that the investment cost for a candidate unit, which selected by the expansion plan, is made at the beginning of the stage in which it goes into service. On the other side, and the salvage value is calculated at the end of the planning horizon^1^.

#### Operating and maintenance cost

This term of the objective function is the generation operation and maintenance costs for existing and new candidate units and assumed to occur in the middle of the corresponding planning stage which is calculated as follows:

5 $$ M\left( {X_{t} } \right) = \mathop \sum \limits_{y = 0}^{s - 1} \left[ {\left( {1 + i} \right)^{{ - \left( {tc + 0.5 + y} \right)}} \times \mathop \sum \limits_{k = 1}^{N} \left[ {FOM_{k} \times X_{t,k} + VOM_{k} \times G_{t,k} } \right]} \right] $$

where, $X_{t}$ and $G_{t,k }$ are the capacity and the expected energy produced for all existing and selected candidate units of each type $k$ in stage $t$; $FOM_{k}$ and $VOM_{k}$ are fixed and variable operating and maintenance (O&M) costs, respectively. The fixed cost of each generation unit is calculated based on its capacity (kW), while the variable cost is proportional to expected energy produced by each generation unit in each stage. Therefore, proper calculation of the expected energy produced by each generation unit is crucial to provide more accurate estimation of the variable costs.

The expected energy produced by each generation unit in the system can be determined by the calculation of the expected energy not served (EENS). The EENS, which is one of reliability indies, can be calculated by conventional method or probabilistic production cost simulation methods to obtain cost modeling. The conventional method is composed of, establishing capacity outage probability table, load probability table and margin between them to calculate the reliability indies such as EENS, and LOLP^7,38^. This method provides a relatively simple approach for EENS calculation but take more computation time, so that will not be suitable for GEP with the large-scale size problem. Therefore, another relative computational speed and solution quality of six different probabilistic production cost simulation methods have been presented for expected energy produced as in^3,39^. In this context, the comparison results have been implied that the probabilistic production cost simulation using the equivalent energy function (EEF) method is more accurate and takes less computation time.

#### Expected energy not served cost ($O\left( {X_{t} } \right))$

There are several reliability indices such as EENS, LOLP and LOLE. The EENS reflects the reliability status of a power system better than other indices. In this context, customer satisfaction with better supply will greatly influence the utility’s competitive ability. Hence, continuously energy supplying, which indicates better system reliability, can achieve customer satisfaction. On the other hand, each generating may be not available at any time depending on its forced outage rate (FOR). So that, the EENS cannot be made zero, but should be minimized as a cost term which can be formulated as follows:

6 $$ O\left( {X_{t} } \right) = \sum\limits_{y = 0}^{s - 1} {\left[ {\left( {1 + i} \right)^{{ - \left( {tc + 0.5 + y} \right)}} \times EENS_{t} \times CEENS} \right]} $$

where, $$CEENS$$ is the cost of EENS in $/MWh. It is assumed that the cost occurs in the middle of the corresponding stage. However, the objective function for solving reliability constrained GEP problem is equivalent to find the optimum expansion planning over planning horizon that minimizes total cost, including capital investment, operation and maintenance, and outage cost under several constraints calculated as follows:

7 $$ Ob = \sum\limits_{t = 1}^{T} {\left[ {I\left( {u_{t} } \right) + M\left( {X_{t} } \right) + O\left( {X_{t} } \right) - SV\left( {u_{t} } \right)} \right]} $$

### Constraints

Several constraints must be considered during the expansion planning process which represented as follows:

- *Upper construction limit*: the number of each generation technology has committed that must satisfy the maximum construction number at stage *t* as follows:
  8 $$ 0 \le u_{t} \le U_{max,t} $$
  where, $U_{max,t}$ is the maximum construction number of each generation type at stage $t$.
- Spinning reserve constraint
  The forecasted load demand and capacity reserve margin constraint must be met by the existing and new selected candidate units, which is represented as follows:
  9 $$ \left( {1 + SR_{min} } \right) \times LD_{t} \le \mathop \sum \limits_{k = 1}^{N} X_{t,k} \le \left( {1 + SR_{max} } \right) \times LD_{t} $$
  where, $X_{t,k}$ is the total capacity of existence and new units, $LD_{t}$ is the peak load demand in the stage t, $SR_{min}$ and $SR_{max}$ minimum and maximum required reserve capacity in stage t, respectively.
- Fuel mix ratio
  Selection of candidate units for expansion planning must be a limited ratio of each technology to all the existing units’ capacity that is expressed as following:
  10 $$ FR_{min}^{j} \le \frac{{X_{t,j} }}{{\mathop \sum \nolimits_{k = 1}^{N} X_{t,k} }} \le FR_{max}^{j} $$
  where, $FR_{min}^{j}$ , and $FR_{max}^{j}$ are lower and upper bounds of jth fuel type mix ratio in stage t, $X_{t,j}$ , capacity of fuel type j in stage t.
- Reliability constraint:
  The existing and new candidate units must satisfy the reliability criterion to maintain supplying energy continuously. LOLP is another reliability index to represent the system robustness in response to any contingencies.
  11 $$ LOLP\left( {X_{t} } \right) \le \varepsilon $$
  where, $\varepsilon$ is the reliability criterion expressed in LOLP that less or equal than $\varepsilon$ in each stage of the planning horizon.

### The equivalent energy function method

**Figure 1:**
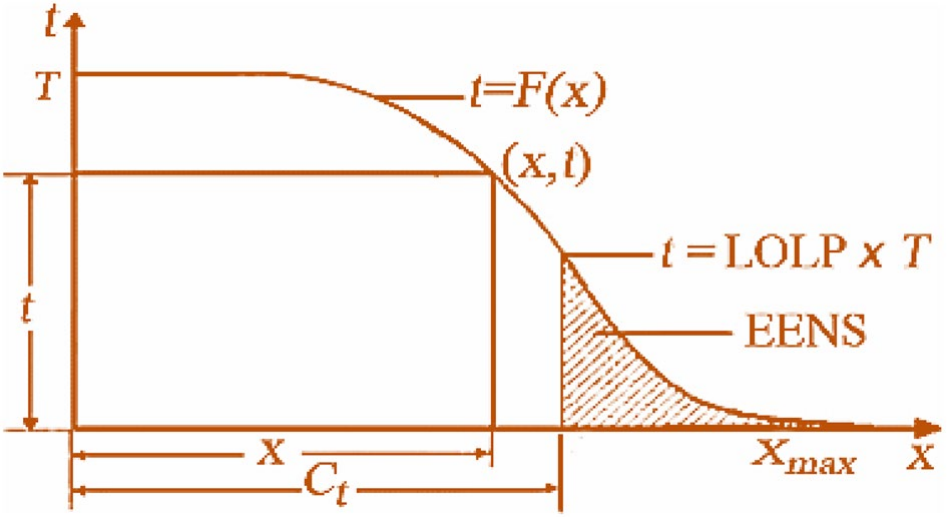
Typical LDC.

As mentioned before, the calculation of expected energy produced by each generation unit, and reliability indices calculation are very important issues for solving GEP problem. For that purpose, probabilistic production simulation (PPS) methods have been widely used for such calculations. The PPS model based on EEF method has been established to analyze the impacts and benefits of efficiency power plant as in^40^. Also, EEF has been applied for estimating the EENS, LOLP, and expected energy produced for solving GEP problem as^9,33,35^ which is dependent on the system load duration curve (LDC) within a period of T as it is shown in Fig. 1. Then, the LDCs probability distribution is $p = f\left( x \right) = F\left( x \right)/T$.

The $x$ axis is divided into sections with lengths of $\Delta x$, the discrete energy function can be defined as follows:

12 $$ E\left( J \right) = \mathop \smallint \limits_{x}^{x + \Delta x} F\left( x \right)dx = T\mathop \smallint \limits_{x}^{x + \Delta x} f\left( x \right)dx $$

where, $J = \left[ {x/\Delta x} \right] + 1$ is an integer, $E\left( J \right)$ is the energy that corresponds to a section of LDC from $x$ to $x + \Delta x$. The corresponding discrete value of the system load $X_{max}$ is $N_{E} = \left[ {X_{max} /\Delta x} \right] + 1$. Therefore, the total energy consumed by the system can be calculated as follows:

13 $$ E_{D} = \mathop \smallint \limits_{0}^{{X_{max} }} F\left( x \right)dx = \mathop \sum \limits_{j = 1}^{{N_{E} }} E\left( J \right) $$

The FOR of the generation units is considered for the energy function that is represented for random outage of the generation units in EEF method. Suppose the generation unit i has a capacity of $C_{i}$,a FOR of $q_{i}$, and an operational availability of $p_{i} = 1 - q_{i}$. Then, then equivalent load duration curve (ELDC) is:

14 $$ f^{i} \left( x \right) = p_{i} f^{{\left( {i - 1} \right)}} \left( x \right) + q_{i} f^{{\left( {i - 1} \right)}} \left( {x - C_{i} } \right) $$

where, $f^{0} \left( x \right)$ is the probability distribution of the primary LDC. The corresponding function can be defined as:

15 $$ E^{i} \left( J \right) = p_{i} E^{{\left( {i - 1} \right)}} \left( J \right) + q_{i} E^{{\left( {i - 1} \right)}} \left( {J - k_{i} } \right) $$

The above equation is the convolution formula in the EEF method, in which $k_{i} = C_{i} /\Delta x.k_{i}$ is an integer because $\Delta x$ is chosen to be the greatest common factor of all unit capacities. Thus, the expected energy produced of $i$th unit should be:

16 $$ E_{gi} = T. p_{i} \mathop \smallint \limits_{{x_{i - 1} }}^{{x_{i - 1} + C_{i} }} f^{{\left( {i - 1} \right)}} \left( x \right)dx = p_{i} \mathop \sum \limits_{{J = J_{i - 1} + 1}}^{{J_{i} }} E^{{\left( {i - 1} \right)}} \left( J \right) $$

where, $$J_{i - 1} = x_{i - 1} /\Delta x$$ and $$J_{i} = \frac{{x_{i - 1} + C_{i} }}{\Delta x} = J_{i - 1} + k_{i} .$$

All the committed generation units have been sorted in ascending order based on their variable costs. Then, the energy function $E\left( J \right)$ is used to calculate the reliability indices as EENS, and LOLP as follows:

17 $$ EENS = \mathop \sum \limits_{{j > j_{n} }} E^{\left( n \right)} \left( J \right) $$

18 $$ LOLP \cong \frac{{E^{\left( n \right)} \left( J \right) + E^{\left( n \right)} \left( {J_{n} + 1} \right)}}{2T\Delta x} $$

where, n is the number of generating units.

### Proposed GEP model modifications

Because the GEP problem is a high dimensional problem with multiple conflicting objectives and nonlinear constraints which makes complex optimization problem, some modifications have been applied to deal with this problem. These modifications such as Virtual mapping procedure (VMP), penalty factor approach (PFA), and modified of intelligent initial population generation (MIIPG) have been used for facility the GEP problem and improve the effectiveness of the meta-heuristic algorithms as follows:

#### Virtual mapping procedure (VMP)

This mapping procedure transforms each combination of candidate units into a dummy variable for each stage. This dummy variable represents a position of each agent in the search space which is updated at each iteration based on using an algorithm. Thus, the decision variable of each stage is represented by single variable only which needs less memory space. Further, if the mapped variable took part in all related solutions, a small change in the mapped variable will reduce the infeasible solutions^2,29,33^. The steps involved in VMP can be stated as follows:

- Form all the possible combinations of the candidate units.
- For each combination, multiply the number of units with the corresponding capacities and add them to get the total capacity of this combination.
- Arrange the total capacity in ascending order based on operation and maintenance costs.

As a result, a multivariable could be reduced to a single variable, which in this case serves as the decision variable. The array size of the solution to the problem will grow by multiples of 5 because it is assumed that five distinct types of units will be used as choice variables at each stage. However, the decision variables obtained by using VMP are a multiple of the number of stages as the array size becomes 1 instead of 5 for each stage. Thus, a size reduction of 80% for each stage is realized. This reduces the dimensionality and the used memory space while improving the algorithm’s performance.

#### Objective function modification (with PFA)

Because the constraints for GEP problem are nonlinear and more complex, obtaining feasible solutions is more difficult. However, the constrained problem can be transformed into an unconstrained problem by using the PFA, which is common for all the meta-heuristic techniques. Thus, the infeasible solutions are avoided in the subsequent iterations by adding proportional penalty values of each violated constraint to the objective function. The objective function with the PFA is given as the fitness function cost (FC):

19 $$ FC_{i} = \left[ {Ob_{i} + \alpha \times \left( {\sum p_{1} + \sum p_{2} + p_{3} } \right)} \right] $$

where, $FC_{i}$ is the objective function modified by PFA of $i$ th individual;$Ob_{i}$ is the objective function value without addition penalty factors for any constraints which is expressed in Eq. (7);$\alpha$ is the penalty factor for the constraints validated;$p_{1}$ is the violation amount of the constraint of spinning reserve margin;$p_{2}$ is the violation amount of the constraint of fuel mix ratio, LOLP;$p_{3}$ is the violation amount of the constraint of LOLP. The penalty factor value must be greater than the total cost with several times to distinguish the feasible and infeasible solutions. When the penalty factor value is small compared with the total costs, the output solution may be infeasible which the results in^9,33^ are the case since their acquired reliability constraint is violated their permissible ranges.

#### Modification of initial population generation (MIIPG)

The first step in any optimization problem is the creation of several populations randomly. Then, the position of each agent is updated according to the performed algorithm. Thus, many of initial solutions may be infeasible due to large search space and complex constraints that affected the convergence and performance of the algorithms. Therefore, the creation of initial population is incorporated to decrease the search space. The minimum and maximum cumulative capacity is considered in this procedure as having been made available in the earlier stages. As a result, the following formula can be used to determine the minimum and maximum capacity needed for each stage depending on reserve margin:

20 $$ CAP_{min,t} = \left( {1 + SR_{min} } \right) \times LD_{t} - CAP_{min,t - 1} $$

21 $$ CAP_{max,t} = \left( {1 + SR_{max} } \right) \times LD_{t} - CAP_{max,t - 1} $$

where, $CAP_{min,t}$, and $CAP_{max,t}$ represent the minimum and maximum capacities needed for a stage *t* based on forecasted load demand and reserve margin. The preceding stage's minimum and maximum capacities, $CAP_{min,t}$, and $CAP_{max,t}$, are calculated as the total of the capabilities of the selected units and existence capacities. Their values are never constant, but rather change with each possible combination that is chosen as in^2^.

## Honey badger algorithm for reliability constrained dynamic GEP

One approach of obtaining the optimal solution of the GEP problem is through the modern meta-heuristic optimization algorithms. Most of these algorithms are based on natural phenomena, where a fitness function is used as an indicator for the distance from the optimal solutions. The advantages of reducing array size by using VMP, converting the constrained problem into an unconstrained problem by using PFA. Also, the solution space reduction can be utilized by using MIIPG and implanted for solving GEP problem. For solving the proposed reliability constrained GEP problem, a novel honey badger algorithm (HBA) is developed. It is a meta-heuristic search algorithm inspired from the intelligent foraging behavior of honey badger to reach its prey. In HBA, the dynamic search behavior of honey badger with digging and honey finding approaches is formulated into exploration and exploitation phases^41^. It prefers to stay alone in self-dug holes and meet the other badgers only to mate. Because of their courageous nature, it can be attacked by even much larger predators when it cannot escape. Also, it can climb on trees for reaching bird nests and beehives for food. A honey badger locates its prey by smelling mouse skills and digging or follows the honey guide bird, which can locate the hives but cannot get honey. The first method of honey badger to reach its food source is called digging mode while the second method is called honey mode. The first mode is executed by honey badger alone, but the second strategy is executed with the help of other birds to locate the hives. The second mode phenomena lead to a relationship between the two which are enjoying the reward of teamwork. However, HBA has dynamic search modes, because of its ability to maintain the trade-off balance between exploration and exploitation in the searching process. The mathematical model of HBA is represented as follows:

The first step of the proposed HBA is to initialize the number of honey badger based on the population size number (*N*) and their respective positions as follows:

22 $$ x_{i} = lb_{i} + r_{1} \times \left( {ub_{i} - lb_{i} } \right) $$

where, $x_{i}$ is honey badger position, $lb_{{\text{i}}}$ and $ub_{{\text{i}}}$ are lower and upper limits of each position in the search space, $r_{1}$ is a random number between 0 and 1.

Then, the intensity (In) is defined which is related to concentration, strength of the prey and distance between it and *i*th honey badger. When the smell is strong, the prey will move quickly, and vice versa is true. Calculating the defining intensity is done as follows:

23 $$ In_{i} = r_{2} \times \frac{S}{{4\pi d_{i}^{2} }} $$

24 $$ S = \left( {x_{i} - x_{i + 1} } \right)^{2} $$

25 $$ d_{i} = x_{prey} - x_{i} $$

where, $$r_{2}$$ is a random number; $$ S$$ is source strength; $$d_{i}$$ denotes distance between $$x_{prey}$$ prey and *i*th badger position.

- To guarantee a smooth transition from the exploration phase to the exploitation phase, the density factor ($\alpha$), which regulates time variable randomness, is specified and updated. The following equation shows how this factor lowers with repeated rounds to reduce randomness over time.:
  26 $$ \alpha = C \times \exp \left( {\frac{ - t}{{iter_{max} }}} \right) $$
  where, $iter_{max}$ is a maximum iterations number, C is a constant equal to 2.
- To enhance escaping from local to optima regions, a flag (*F*) is generated which alters the search direction. Thus, agents now have several options for thoroughly exploring the search space. The equation that follows determines it:
  27 $$ F = \left\{ {\begin{array}{*{20}l} 1 \hfill & {if r_{3} \le 0.5} \hfill \\ { - 1} \hfill & {other wise} \hfill \\ \end{array} } \right. $$
  where, $r_{3}$ is the random number between [0,1].
- Then, the agent’s positions are updated where $$ x_{new}$$ is updated according to two phases of digging phase, and honey phase as follows:

In the digging phase, a honey badger performs actions like Cardioid shape which can be simulated as follows:

28 $$ x_{new} = x_{prey} + F \times \beta \times I \times x_{prey} + F \times r_{4} \times \alpha \times d_{i} \times \left| {\cos \left( {2\pi r_{5} } \right) \times \left[ {1 - \cos \left( {2\pi r_{6} } \right)} \right]} \right| $$

where, $\beta$ is the ability of the honey badger to get food which is greater than or equal to 1 (default = 6), $r_{4}$, $r_{5}$, and $r_{6}$ are three different random numbers between 0, and 1.

**Figure 2:**
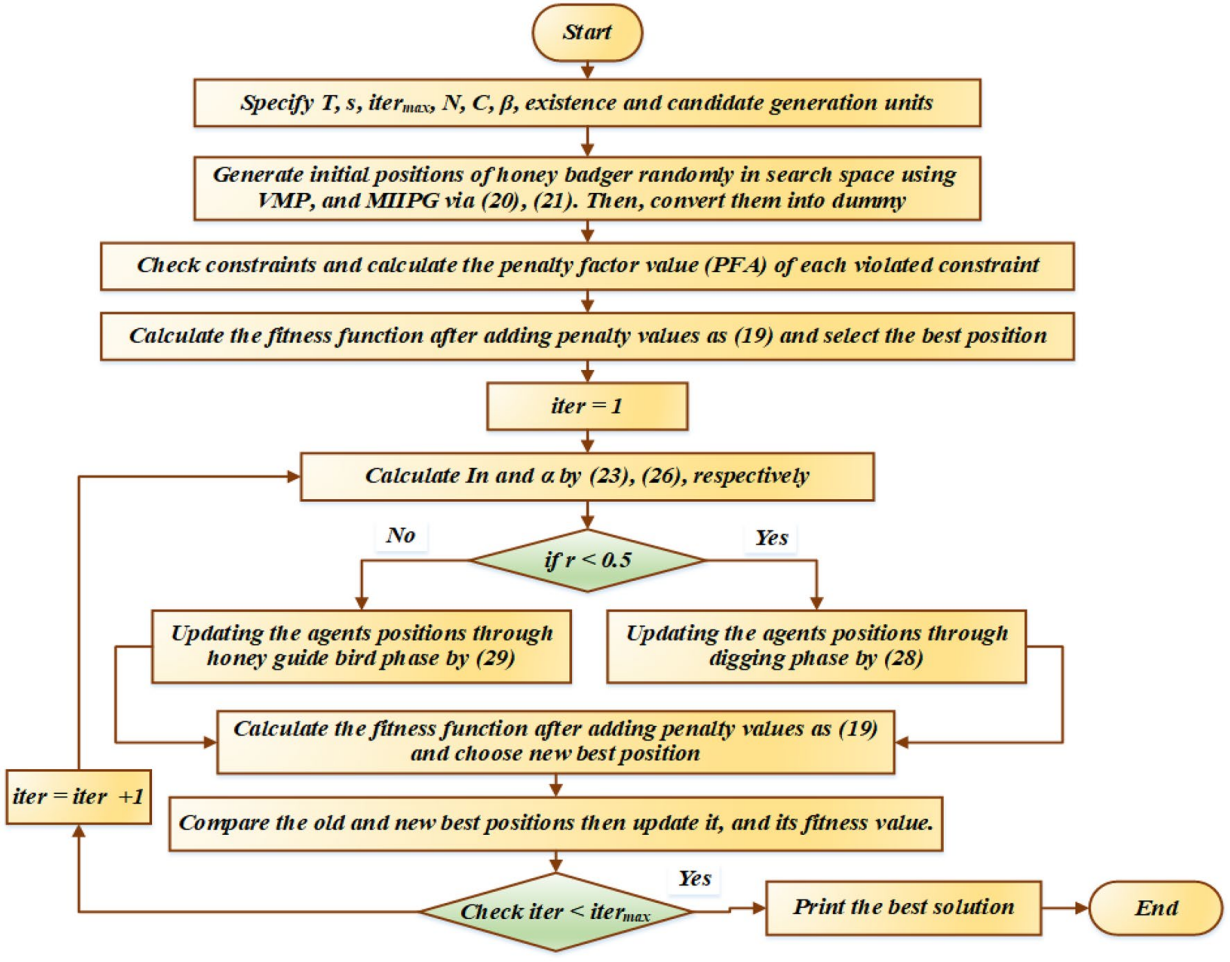
Flowchart for the proposed HBA for solving the GEP problem.

A honey badger follows the honey guide bird to the replicated beehive during the honey phase.

29 $$ x_{new} = x_{prey} + F \times r_{7} \times \alpha \times d_{i} $$

where, $r_{7}$ is a random number between 0 and 1, $d_{i}$ and $\alpha$ are calculated using Eqs. (25) and (26), respectively. The flowchart diagram of the HBA is shown in Fig. 2.

Therefore, a honey badger searches near its prey, and the search is influenced by time variations ($\alpha$). Additionally, two user-defined parameters ($\beta \;and\;C$) have a considerable impact on the HBA performance, thus it is important to choose the parameter values carefully. The optimum parameter values for the suggested algorithm in this situation are ($\beta$ = 6, and $C$ = 2), which are given from ^41^.

Added to that, several modern meta-heuristic optimization algorithms are employed which are crow search algorithm, aquila optimizer, bald eagle search and particle swarm optimization. The GEP problem has been solved using PSO and some modified versions of it as^2,35,42^. Also, CSA is a one of modern optimization tools which is based on crow’s intelligence in storing and retrieving its food in hiding locations^43^. This algorithm is used for the first time to solve the GEP problem, but it has been applied for solving several optimization problems such as economic dispatch^44^, unit commitment^45^, preventive maintenance scheduling^46^, capacitor allocation in distribution systems^47^, and optimal power flow problem^48,49^.

The AO is a one of modern meta-heuristic optimization approach inspired by the natural behavior of the Aquila during the prey capturing process. Aquila has potential for switching of catching a prey process that it is speed and agility. The choice of hunting tactics is also influenced by the hunting circumstances. However, more details about AO is in^50^. The BES algorithm is a novel meta-heuristic optimization algorithm which mimics the hunting process or intelligent behavior of the bald eagle as they search for fish^51^. Hence, the hunting process of the proposed BES is represented in three strategies, namely, selecting search space, searching within the selected search space, and swooping. For the tested systems all constraints presented in Eqs. (7)–(10) are preserved beyond their permissible limits using all optimization algorithms.

## Test system description and results discussion

In this section, the proposed HBA is performed for solving three cases based on the considered planning horizon as:

- Case-1: short-term GEP problems with 6-year planning horizon
- Case-2: long-term GEP problems with 12-year planning horizon
- Case-3: long-term GEP problems with 24-year planning horizon

Each planning horizon consists of two years of stage, making them to be 3, 6, and 12 stages in short and long-term planning horizon. Number of years between the reference date of cost calculations and the first year of study ($t_{0}$) are assumed to be 2 years.

### Test system description

In this paper, the GEP problem is solved by PSO, CSA, AO, BES and HBA. The simulation results are carried out on a test system whose date is reported in Supplementary Material. The test system consists of 15 existing generation units (based on type of fuel used: Oil, Liquid Natural Gas (LNG), coal and Nuclear (Nuc. PWR)) as in Table S.1. While the 5 different new generation technology is selected as candidate units (based on fuel type used: Oil, Liquid Natural Gas (LNG), Coal, and Nuclear (Nuc. PWR and Nuc. PHWR)) as in Table S.2. The forecasted peak load, as in Table S.3 with initial peak load of 5000 MW, and other data of existing and candidate generation units are taken from^9,35^. Different parameters have been used for the same test system at different planning horizons such as^2,9,35^. In this paper, the discount rate is taken 8.5%, LOLP criteria at each stage is considered of 0.01. The lower and upper limits for reserve margin are set at 20% and 50%, respectively. EENS cost is assumed to be 0.05 $/kWh. The lower and upper bounds of capacity mixes by fuel types are 0% and 30% for oil-fired power plants, 0% and 40% for LNG-fired, 20% and 60% for coal-fired, and 30% and 60% for nuclear, respectively. The cost of unserved energy, or EENS, which is calculated by EEF method, is set at 0.05 $/kWh. The initial period is set as two years while the investment cost is assumed to occur in the beginning of the year and the salvage cost is assumed to occur at the end of the planning horizon.

### Parameters for GEP and optimization algorithms

The utilized optimization algorithms of PSO, CSA, AO, BES besides the proposed HBA is handled for solving the short- and long-term capacity expansion planning based on 200 of iteration number, and 60 of population size for 30 simulation runs. In BES algorithm, each population of solution has been updated three times at each iteration where it is updated once for the other applied algorithms. Thus, the population size is 30 for BES only. Moreover, the penalty factor value ($\alpha$), which is equal to 10^15^, is used to penalize the infeasible solutions.

### Numerical results and discussion

By using VMP to create dummy control variables, PFA to convert the constraint problem into unconstrained problem, and MIIPG to decrease the search space, PSO, CSA, AO, BES, and the proposed HBA are carried out for solving the short- and long-term reliability constraint GEP planning horizon.

#### Simulation results for case-1

In case-1, the CSA, AO, BES, PSO, and the proposed HBA are employed for solving reliability constraint GEP over 6-years (3 stages) planning horizon. Table 1 summarizes the optimal results obtained by each applied algorithm for reliability GEP for 6 years planning horizon which are the number of each type of power plants in each stage. The total cost achieved results in terms of the statistical indices of each applied algorithm is recorded in Table 2. The obtained comparative results show that the proposed HBA has achieved the best total costs compared to the other applied algorithms, whereas the improvement of the objective function is equal to 3.2%, 1.1% and 0.081% in costs over the CSA, AO and BES, respectively. Although the best cost of the proposed HBA is equal to that of PSO, the HBA algorithm achieves the standard deviation and standard error of (2.27/12.5) × 10^7^, which are less than their counter parts for CSA, AO, BES, and PSO algorithms with (2.58/14.1) × 10^7^, (2.99/16.4) × 10^7^, (2.53/13.8) × 10^7^, and (3.09 /16.9) × 10^7^ respectively. These findings demonstrate the effectiveness of the HBA for solving the GEP in case-1 compared to the other algorithms.

**Table 1:** Number of newly candidate units in each stage for case 1.

Stage		I					II					III				
Units		oil	LNG	Coal	Nuc. (PWR)	Nuc. (PHWR)	oil	LNG	Coal	Nuc. (PWR)	Nuc. (PHWR)	oil	LNG	Coal	Nuc. (PWR)	Nuc. (PHWR)
Algorithm	CSA	1	4	3	1	0	5	1	0	1	0	0	0	1	1	0
	AO	1	4	3	1	0	1	3	0	1	0	1	1	0	1	0
	BES	1	4	3	1	0	0	4	0	1	0	0	1	0	1	0
	PSO	1	4	3	1	0	0	4	0	1	0	0	0	0	1	0
	HBA	1	4	3	1	0	0	4	0	1	0	0	0	0	1	0

**Table 2:** Statistical results for case-1 (3 stages) 6- years.

Total cost ($)	CSA	AO	BES	PSO	HBA
Best cost*10^8^	67.331	65.924	65.734	65.204	65.204
Average cost*10^8^	70.613	70.511	69.63	68.125	68.56
Worst cost*10^8^	73.464	73.267	71.976	72.265	71.736

**Figure 3:**
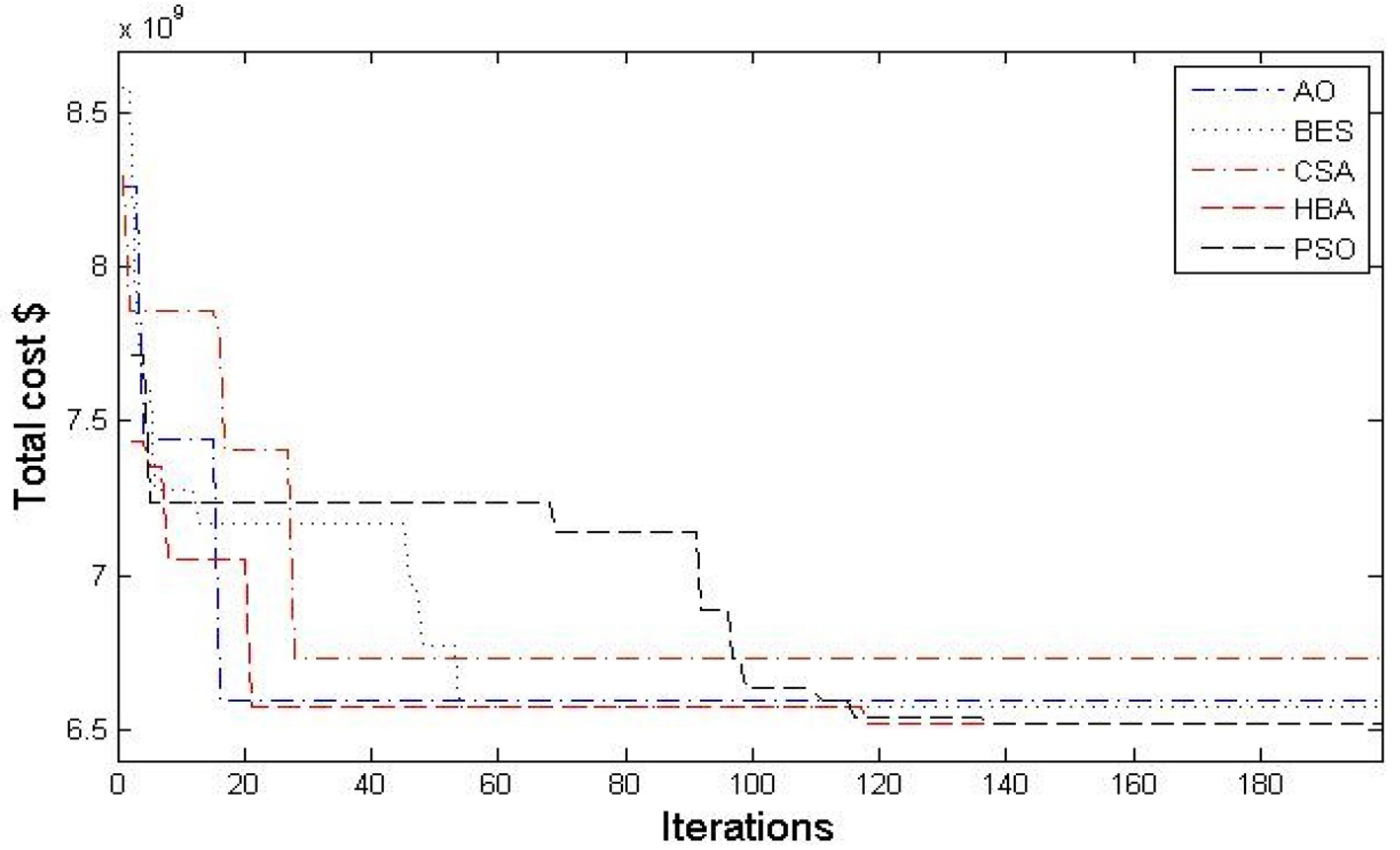
Convergence characteristics of comparison algorithms for case 1.

**Figure 4:**
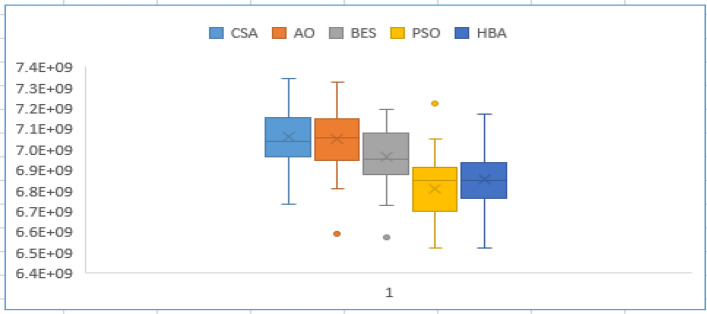
Box chart of variations of runs for case 1.

Moreover, Figs. 3 and 4 describe the convergence curves and box graph of CSA, AO, BES, PSO and the proposed HBA. From Fig. 3, it can be observed that the HBA algorithm shows better convergence compared to the others. From Fig. 4, the proposed HBA shows significant results with the smallest length of the box plot. In addition, it provides the smallest worst objective with 7.1736 × 10^9^ $ where the CSA, AO, BES and PSO achieve 7.3464 × 10^9^, 7.3267 × 10^9^, 7.1976 × 10^9^ and 7.2265 × 10^9^ $, respectively. Also, the reliability criterion of LOLP obtained are shown in Table 3 for each stage which indicates that the reliability criterion is satisfied for each applied algorithm. As shown, both HBA and PSO the smallest LOLP of 0.009787, 0.005251 and 0.008899 for the three stages planning, respectively.

**Table 3:** values of LOLP criterion for case 1.

Stages	CSA	AO	BES	PSO	HBA
I	0.009787	0.009787	0.009787	0.009787	0.009787
II	0.00879	0.007991	0.005251	0.005251	0.005251
III	0.005648	0.00374	0.003686	0.008899	0.008899

#### Simulation results for case-2

**Figure 5:**
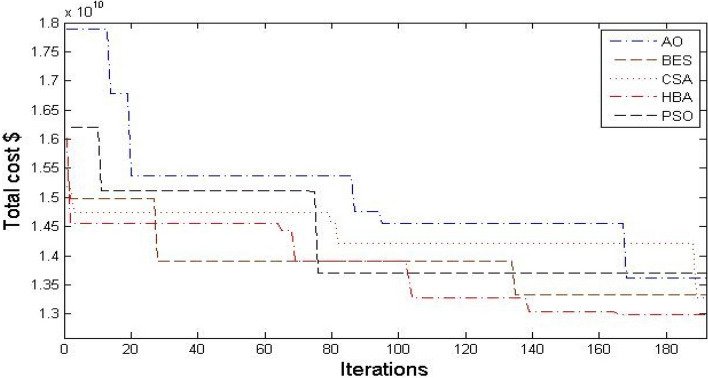
Convergence characteristics of comparison algorithms for case-2.

**Figure 6:**
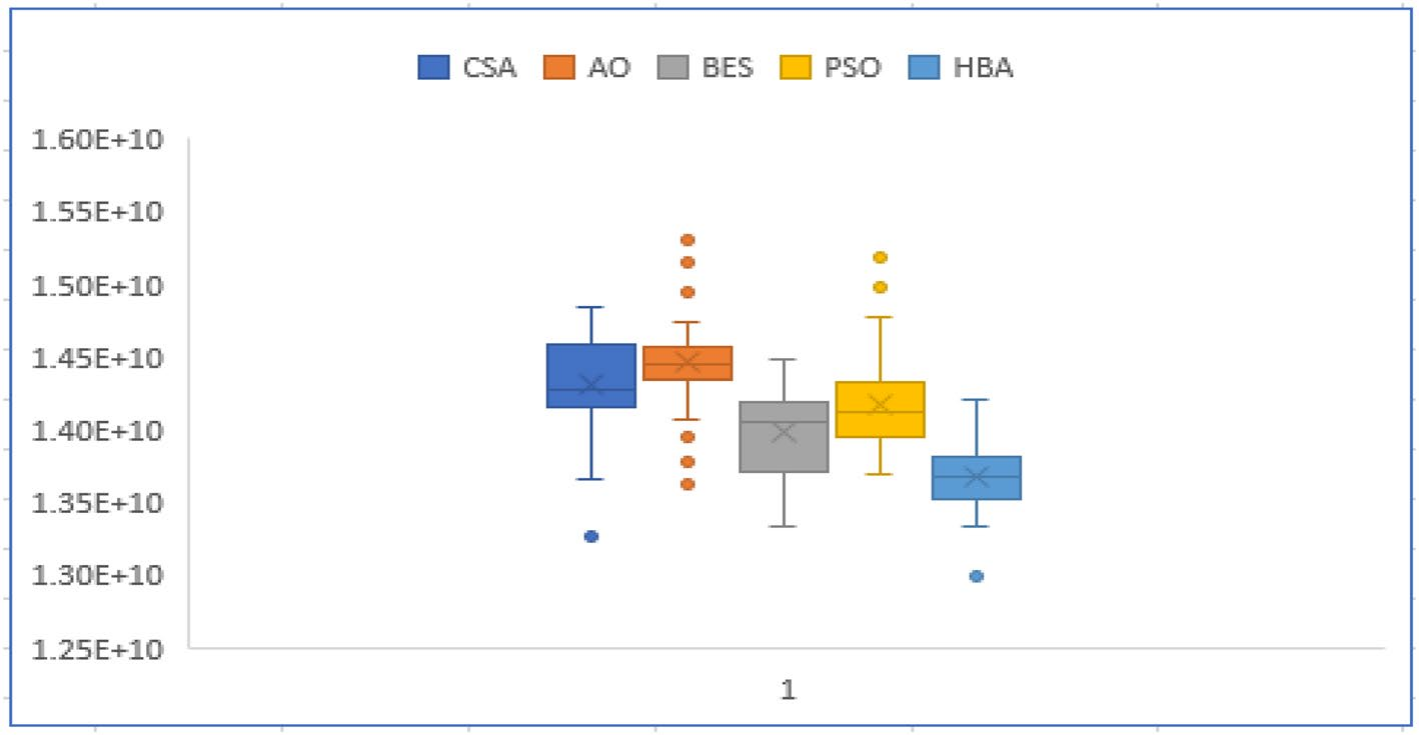
Box chart of variations of runs for case 2.

In this case, a long-term planning horizon 12-year (6 stages) is conducted. CSA, AO, BES, PSO, and the proposed HBA are employed for solving reliability constraint GEP for this case. Table 4 shows the number of new candidate generation units for each stage of the planning horizon. According to the results of case 2, their achieved results in terms of the statistical indices of each applied algorithm is recorded in Table 5. For 12-year planning horizon, it is found that the proposed HBA provides the minimum best total costs and better performance than other algorithms. The proposed HBA has achieved a 2.5%, 4.5%, 2.5%, and 5.16% enhancement in costs over CSA, AO, BES, and PSO, respectively. It should be noted that the performance of PSO algorithms is affected by the planning horizon than others. Figures 5 and 6 show the convergence characteristics and box chart of CSA, AO, BES, PSO and the proposed HBA which ensures the better performance and effectiveness of the proposed HBA. From Fig. 5, the proposed HBA algorithm shows better convergence compared to the others. From Fig. 6, the proposed HBA shows significant superiority as follows:

- The proposed HBA algorithm provides the smallest average objective with 1.3675 × 10^10^$.
- The proposed HBA algorithm provides the smallest worst objective with 1.4211 × 10^10^$.
- The proposed HBA algorithm provides the smallest standard error and standard deviation with (4.43/24.3) × 10^7^$, compared to (6.32/3.46) × 10^7^, (6.42/3.51) × 10^7^, (6.87/3.76) × 10^7^, (5.57/3.05) × 10^7^ $ for the CSA, AO, BES, PSO algorithms.

**Table 4:** Number of newly candidate units in each stage for case 2.

Stage	Algorithm	oil	LNG	Coal	Nuc. (PWR)	Nuc. (PHWR)	Stage	Algorithm	oil	LNG	Coal	Nuc. (PWR)	Nuc. (PHWR)
Stage I	CSA	1	2	3	2	0	Stage IV	CSA	1	2	2	0	0
	AO	5	2	2	2	0		AO	3	2	1	0	0
	BES	2	3	2	2	0		BES	0	2	2	1	0
	PSO	2	2	3	2	0		PSO	2	3	0	0	0
	HBA	1	4	3	1	0		HBA	0	2	2	1	0
Stage II	CSA	3	4	0	0	0	Stage V	CSA	1	0	0	1	0
	AO	2	3	1	0	0		AO	0	2	0	1	0
	BES	2	4	0	0	0		BES	5	0	0	0	0
	PSO	2	3	0	1	0		PSO	2	0	0	1	0
	HBA	0	4	0	1	0		HBA	1	2	0	0	0
Stage III	CSA	1	2	0	1	0	Stage VI	CSA	1	1	1	0	0
	AO	0	0	1	1	0		AO	3	0	2	0	0
	BES	0	0	1	1	0		BES	5	1	0	0	0
	PSO	0	1	2	0	0		PSO	3	3	0	0	0
	HBA	0	1	0	1	0		HBA	3	0	1	0	0

**Table 5:** Statistical results for case-2.

Total cost $	PSO	CSA	AQ	BES	HBA
Best cost *10^9^	13.702	13.327	13.620	13.335	12.996
Average cost *10^9^	14.179	14.306	14.473	13.982	13.675
Worst cost *10^9^	15.179	14.45	15.319	14.477	14.211

In this context, the values of LOLP criterion of each stage, which must be satisfied, are also shown in Table 6. As shown, the reliability criterion is satisfied for each applied algorithm.

**Table 6:** Values of LOLP criterion for case 2.

Stages	CSA	AO	BES	PSO	HBA
I	0.009599	0.004759	0.006968	0.006274	0.009787
II	0.008322	0.00566	0.009314	0.003352	0.005251
III	0.001558	0.003678	0.005979	0.001958	0.003686
IV	0.002614	0.006715	0.002589	0.006253	0.001543
V	0.002958	0.002033	0.003092	0.004534	0.001802
VI	0.003027	0.000802	0.001573	0.00095	0.001897

#### Simulation results for case-3

**Figure 7:**
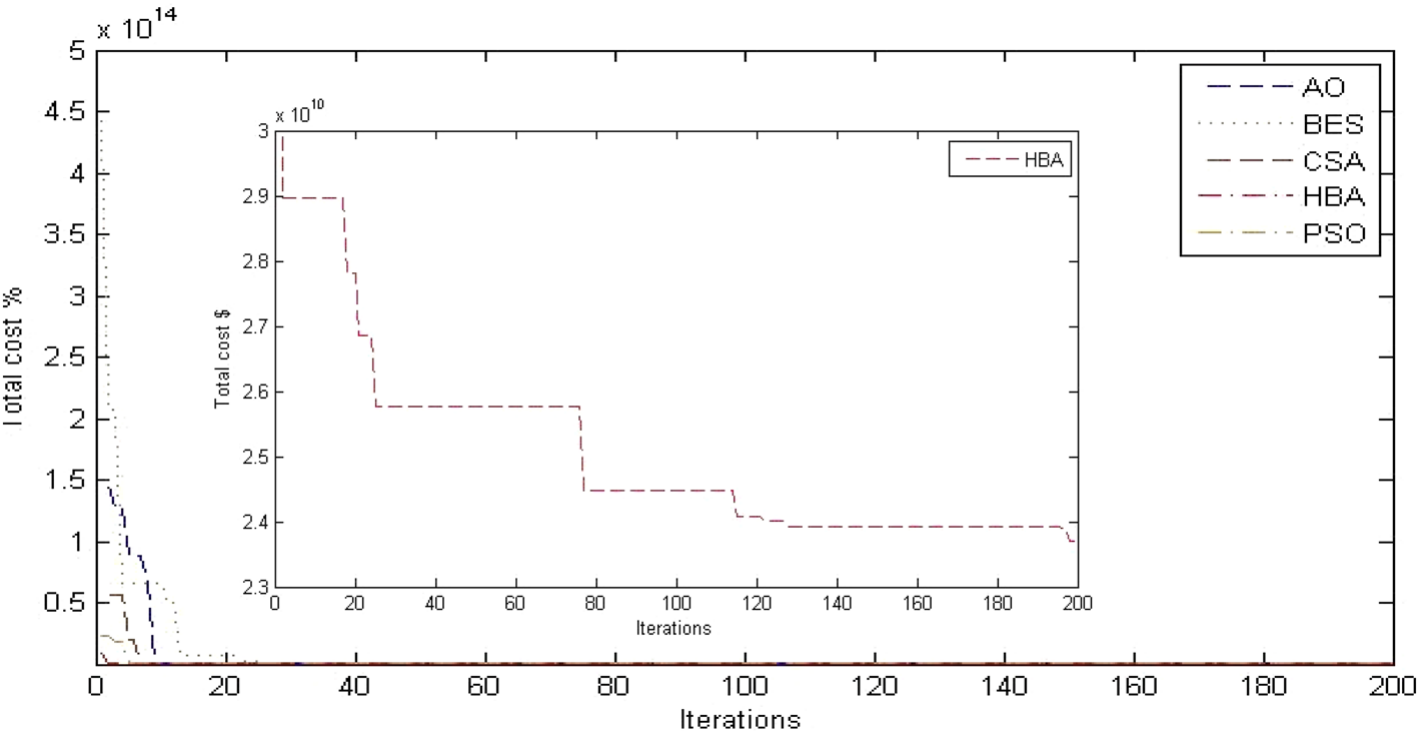
Convergence characteristics of comparison algorithms for case 3.

**Figure 8:**
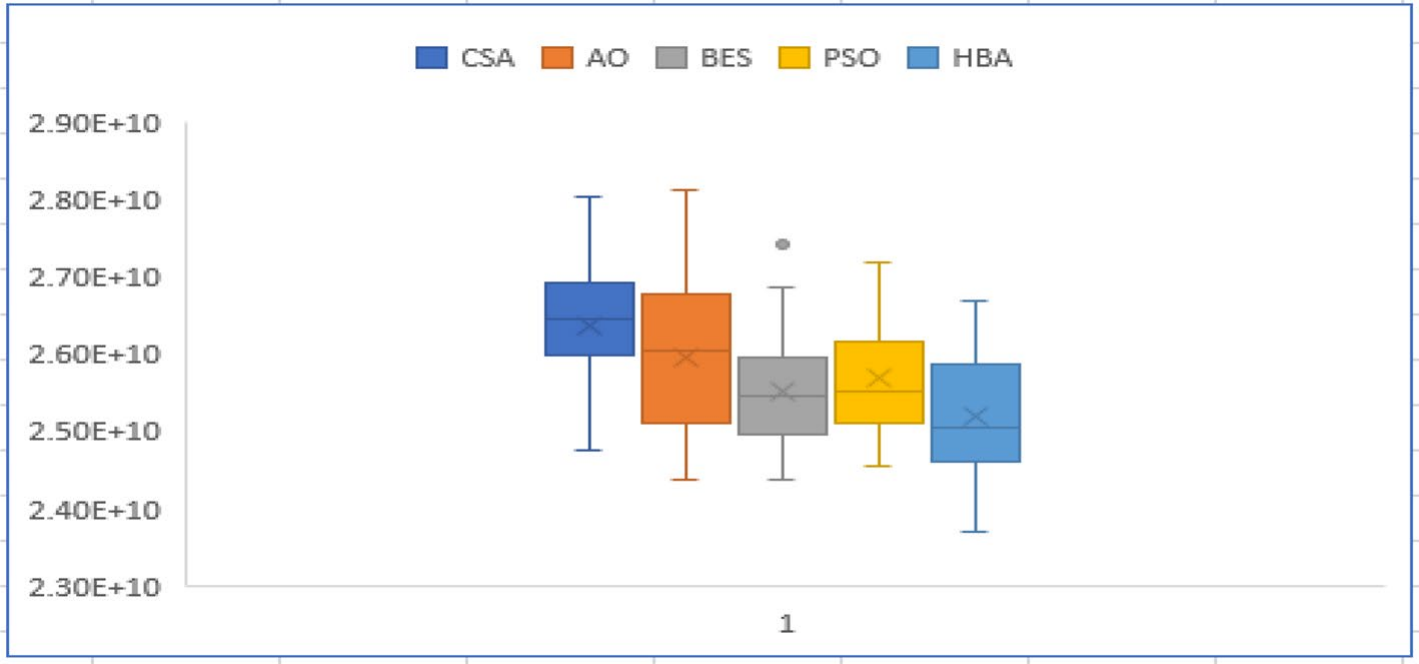
Box chart of variations of runs for case 3.

In this case, a long-term planning horizon 24-year (12 stages) is conducted. CSA, AO, BES, PSO, and the proposed HBA are employed for solving reliability constraint GEP for this case. Table 7 shows the number of new candidate generation units for each stage of the planning horizon. According to the results of case 2, their achieved results in terms of the statistical indices of each applied algorithm is recorded in Table 8. The obtained results comparison shows that the proposed HBA has achieved 4.2%, 2.72%, 2.7%, and 3.4% over other applied algorithms. Thus, the HBA achieves the optimum reliability constrained GEP with minimum total cost and better performance for the long-term planning horizon. Figures 7 and 8 show the convergence characteristics and box chart of CSA, AO, BES, PSO and the proposed HBA which ensures the better performance and effectiveness of the proposed HBA. From Fig. 7, the proposed HBA algorithm shows better convergence compared to the others. From Fig. 8, the proposed HBA shows significant superiority as follows:

- The proposed HBA algorithm provides the smallest average objective with 2.4953 × 10^10^ $.
- The proposed HBA algorithm provides the smallest worst objective with 2.6569 × 10^10^ $.
- The proposed HBA algorithm provides standard error and standard deviation with (1.33/73.1) × 10^8^ $, respectively which are smallest than that obtained by CSA, AO, BES, and PSO algorithms as (1.34/7.35), (1.48/8.13), (1.86/10.2), (1.34/7.35) × 10^8^ $, respectively.

**Table 7:** Number of newly candidate units in each stage for case 3.

Stages	Algorithm	Candidate units					Stages	Algorithm	Candidate units				
		Oil	LNG	Coal	Nuc. (PWR)	Nuc. (PHWR)			Oil	LNG	Coal	Nuc. (PWR)	Nuc. (PHWR)
I	CSA	2	3	2	2	0	VII	CSA	1	3	1	0	1
	AO	1	4	2	2	0		AO	1	0	2	0	0
	BES	2	3	2	2	0		BES	2	2	1	0	1
	PSO	1	4	3	1	0		PSO	1	1	1	0	1
	HBA	1	4	2	2	0		HBA	0	0	0	1	0
II	CSA	1	3	1	1	0	VIII	CSA	1	1	1	1	0
	AO	0	2	1	1	0		AO	3	1	1	0	0
	BES	1	2	1	1	0		BES	2	0	1	1	0
	PSO	5	0	1	2	0		PSO	2	0	0	1	0
	HBA	1	2	1	1	0		HBA	3	2	1	0	0
III	CSA	1	1	0	0	0	IX	CSA	4	0	0	0	0
	AO	2	1	2	0	0		AO	5	0	2	0	0
	BES	1	0	0	0	1		BES	1	1	2	1	0
	PSO	1	1	0	0	0		PSO	1	2	2	1	0
	HBA	0	0	2	0	0		HBA	0	1	0	0	1
IV	CSA	3	2	2	1	0	X	CSA	1	2	2	2	0
	AO	0	2	1	1	0		AO	0	2	1	1	0
	BES	1	1	2	1	0		BES	0	0	0	0	0
	PSO	3	2	2	0	0		PSO	1	1	1	1	0
	HBA	1	2	2	1	0		HBA	1	0	0	2	0
V	CSA	1	0	0	1	1	XI	CSA	1	1	1	1	0
	AO	3	1	0	1	0		AO	0	2	2	1	0
	BES	5	0	0	0	0		BES	3	0	1	0	2
	PSO	1	0	0	2	0		PSO	0	3	2	1	0
	HBA	0	1	0	1	0		HBA	0	2	1	0	1
VI	CSA	0	0	0	0	0	XII	CSA	5	3	2	0	1
	AO	1	0	1	1	0		AO	2	2	3	1	0
	BES	1	0	0	1	0		BES	2	3	2	1	0
	PSO	0	4	0	0	0		PSO	0	2	2	0	2
	HBA	1	4	0	0	0		HBA	2	1	1	0	2

**Table 8:** Statistical results for case 3.

Total cost $	PSO	CSA	AQ	BES	HBA
Best cost*10^9^	24.541	24.753	24.369	24.366	23.706
Average cost*10^9^	25.687	26.369	25.95	25.521	24.953
Worst cost*10^9^	27.193	28.054	28.125	27.434	26.569

In addition to, the reliability criterion is satisfied for each stage which the values of LOLP are given in Table 9. From the three cases studied of short- and long-term planning horizon, the proposed HBA algorithm obtains the optimum reliability expansion planning with minimum objective function and satisfied constraints better than CSA, AO, BES, and PSO algorithms.

**Table 9:** Values of LOLP criterion for case 3.

Stages	CSA	AO	BES	PSO	HBA
I	0.006968	0.004243	0.006968	0.009787	0.004243
II	0.002129	0.005301	0.005424	0.001211	0.003268
III	0.005979	0.001217	0.008398	0.003678	0.004703
IV	0.000687	0.001444	0.005643	0.002545	0.001304
V	0.000192	0.000284	0.006662	0.000495	0.000945
VI	0.002008	0.000122	0.007035	0.000138	0.000179
VII	8.47E−05	0.000116	0.000566	3.24E−05	0.000326
VIII	0.000167	0.000527	0.0015	0.000294	0.000596
IX	0.000308	9.18E−05	0.00011	7.22E−06	0.000622
X	1.52E−05	0.000108	0.006699	1.49E−05	0.001041
XI	2.84E−05	4.76E−05	0.004568	2.64E−06	0.001497
XII	8.26E−07	3.33E−06	0.000487	3.9E−07	0.000695

To provide a comparison with literature work, Table 10 shows and analyzes the main differences between the presented study and other previously works. As shown, the presented study based on the HBA can achieve the highest reserve margin reaching 60%. In this regard, same margin has been addressed via the differential evolution in^34^ but the proposed study achieved greater reduction the investment costs. Moreover, the presented study based on the HBA addressed three different planning horizons of 6, 12 and 24 years. Otherwise the loss of load probability has been greatly minimized to 0.01 using the presented study based on the HBA and the elitist Non-dominated Sorting Genetic Algorithm version II^29^.

**Table 10:** Main differences between the presented study and other previously works.

Ref. number	Methodology	lolp	Reserve margin (%)	Planning horizon (years)	Total costs *109 $
^33^	shuffled frog leaping algorithm and genetic algorithm	0.0027	20–50	10	10.93
				20	19.68
^9^	modified shuffled frog leaping algorithm (MSFLA)	0.0027	20–50	12	12.93
				24	22.51
^29^	elitist Non-dominated Sorting Genetic Algorithm version II	0.01	20–40	6	12.6275
^2^	The PSO and its variants	–	20–40	6	12.009
				14	21.865
^34^	Multi-objective differential evolution	0.0042	UP TO 60%	6	82.963
Presented Study	HBA	0.01	50% and 60%	6	6.5204
				12	12.996
				24	23.706

## Conclusions

This paper presents a novel honey badger algorithm (HBA) for solving the proposed reliability constrained Generation expansion planning (GEP) problem. In the GEP problem, a multi-stage model with reliability constrained is presented to minimize the total costs over the planning horizon maintaining several practical constraints of the spinning reserve, the fuel mix ratio and Loss of Load Probability. Also, the Virtual mapping procedure, penalty factor approach, and the modified of intelligent initial population generation are incorporated to the HBA to decrease the search space and reduce the computational time. Besides the proposed HBA, four modern meta-heuristic optimization algorithms were applied in three test cases to solve the short- and long-term reliability constraint GEP problem. They are crow search algorithm, aquila optimizer, bald eagle search and particle swarm optimization. The proposed HBA was successfully applied to reliability constrained GEP problems, with results that outperform CSA, AO, BES, and PSO in term of best, average, worst, standard error, and standard deviation. The proposed HBA achieves an order of magnitude of improvement for short- and long- term expansion planning than other algorithms. Future studies will be made to incorporate uncertainties such as load demand, and outage of generation units within GEP problem.

### Supplementary Information

Supplementary Tables.

## Data Availability

All required data are involved in the text.
