# Supplementary material for: Reliability constrained dynamic generation expansion planning using honey badger algorithm
Source: Sci Rep. 2023 Oct 5;13:16765. doi: 10.1038/s41598-023-43622-9 (PMC10556132; doi:10.1038/s41598-023-43622-9)
Supplement: Supplementary file 1 — Supplementary Tables. [file 41598_2023_43622_MOESM1_ESM.docx]

**Supplementary material**

Table S.1 Technical and economic data of existing plants

| Name | No. of units | Unit capacity (GW) | FOR% | Operating cost ($/kWh) | Fixed O&M cost ($/kW-Mon) |
| --- | --- | --- | --- | --- | --- |
| Oil#1 | 1 | 0.2 | 7.0 | 0.024 | 2.25 |
| Oil#2 | 1 | 0.2 | 6.8 | 0.027 | 2.25 |
| Oil#3 | 1 | 0.15 | 6.0 | 0.030 | 2.13 |
| LNG G/T #1 | 3 | 0.05 | 3.0 | 0.043 | 4.52 |
| LNG C/C #1 | 1 | 0.4 | 10.0 | 0.038 | 1.63 |
| LNG C/C #3 | 1 | 0.4 | 10.0 | 0.040 | 1.63 |
| LNG C/C #4 | 1 | 0.45 | 11.0 | 0.035 | 2.00 |
| Coal #1 | 2 | 0.25 | 15.0 | 0.023 | 6.65 |
| Coal #2 | 1 | 0.5 | 9.0 | 0.019 | 2.81 |
| Coal #3 | 1 | 0.5 | 8.5 | 0.015 | 2.81 |
| Nuc (PWR) | 1 | 1 | 9.0 | 0.005 | 4.94 |
| Nuc (PWR) | 1 | 1 | 8.8 | 0.005 | 4.63 |

Table S.2 Technical and economic data of candidate plants

| New units | $U_{max}$ | Capacity (MW) | FOR % | Operating cost ($/kWh) | Fixed O&M cost ($/kW-Mon) | Capital cost ($/kW) | Lifetime (yrs) |
| --- | --- | --- | --- | --- | --- | --- | --- |
| Oil | 5 | 200 | 7.0 | 0.021 | 2.20 | 812.5 | 25 |
| LNG C/C | 4 | 450 | 10.0 | 0.035 | 0.90 | 500.0 | 20 |
| Coal (bit) | 3 | 500 | 9.5 | 0.014 | 2.75 | 1062.5 | 25 |
| Nuc. (PWR) | 3 | 1000 | 9.0 | 0.004 | 4.60 | 1625.0 | 25 |
| Nuc. (PHW) | 3 | 700 | 7.0 | 0.003 | 5.50 | 1750.0 | 25 |

Table S.3 Forecasted peak demand.

| Stage | I | II | III | IV | V | VI | VII | VIII | IX | X | XI | XII |
| --- | --- | --- | --- | --- | --- | --- | --- | --- | --- | --- | --- | --- |
| Peak load (MW) | 7000 | 9000 | 10000 | 12000 | 13000 | 14000 | 15000 | 17000 | 18000 | 20000 | 22000 | 24000 |
